# A longitudinal residential relocation study of changes in street layout and physical activity

**DOI:** 10.1038/s41598-021-86778-y

**Published:** 2021-04-08

**Authors:** Gavin R. McCormack, Mohammad Javad Koohsari, Jennifer E. Vena, Koichiro Oka, Tomoki Nakaya, Jonathan Chapman, Ryan Martinson, Graham Matsalla

**Affiliations:** 1 Department of Community Health Sciences, Cumming School of Medicine, University of Calgary, 3280 Hospital Drive, NW, Calgary, Alberta T2N 4Z6 Canada; 2 Faculty of Kinesiology, University of Calgary, Calgary, Canada; 3 School of Architecture, Planning and Landscape, University of Calgary, Calgary, Canada; 4 Faculty of Sport Sciences, Waseda University, Tokorozawa, Japan; 5 Behavioural Epidemiology Laboratory, Baker Heart and Diabetes Institute, Melbourne, Australia; 6 Melbourne School of Population and Global Health, The University of Melbourne, Melbourne, Australia; 7 CancerCare Alberta, Alberta Health Services, Alberta, Canada; 8 Graduate School of Environmental Studies, Tohoku University, Sendai, Japan; 9 Transportation Planning, Transportation Department, The City of Calgary, Alberta, Canada; 10 Toole Design Group, Calgary, Alberta Canada; 11 Mental Health Promotion and Illness Prevention Alberta Health Services, Alberta, Canada

**Keywords:** Psychology and behaviour, Risk factors

## Abstract

Few longitudinal residential relocation studies have explored associations between urban form and physical activity, and none has used the Space Syntax theory. Using a Canadian longitudinal dataset (n = 5944), we estimated: (1) differences in physical activity between non-movers, and those relocating to neighbourhoods with less or more integrated street layouts, and; (2) associations between changes in street layout integration exposure and differences in physical activity. Adjusting for covariates, we found relative to non-movers, those who moved to more integrated neighbourhoods undertook significantly (p < .05) more leisure walking (27.3 min/week), moderate-intensity (45.7 min/week), and moderate-to-vigorous intensity physical activity (54.4 min/week). Among movers, a one-unit increase in the relative change in street integration exposure *([Street integration at follow-up—street integration at baseline]/street integration at baseline*) was associated with a 7.5 min/week increase in leisure walking. Our findings suggest that urban design policies that improve neighbourhood street integration might encourage more physical activity in adults.

## Introduction

Land use patterns (e.g., an arrangement of destinations, mix of uses, distribution of recreational opportunities), urban design features (e.g., safety, aesthetics, friendliness, and vibrancy), and transportation systems (e.g., road, sidewalk/pathway and other transportation infrastructure, connections, and linkages) are linked to health via the built environment’s influence on physical activity^1^. Despite methodological limitations, cross-sectional evidence suggests that neighbourhood built characteristics are associated with physical activity in adults^2^. Notably, pedestrian and street connectivity, land use and destination diversity, density, greenspaces, buildings, and walkability are associated with physical activity^3–7^. During the last two decades, natural experiments and residential relocation studies exploring associations between changes in the built environment and physical activity have emerged, demonstrating tentative findings^8–10^. Evidence from retrospective longitudinal studies shows consistent associations between the built environment and transport walking following residential relocation^9^. Less consistent evidence from retrospective longitudinal studies exists for associations between the built environment and recreational physical activity, public transport use, and cycling following residential relocation^9^. Some evidence from prospective longitudinal residential relocation studies has also found positive associations between walkability and physical activity^9^.

Like population or residential density and land use or destination mix, street layout is an important built environment feature that influences physical activity^3–7, 11^, and in particular walking^12, 13^. Street connectivity reflects the directness of routes linking destinations and the ease at which someone can travel between destinations^14–16^. Neighbourhoods with higher street connectivity often have grid-like street patterns, short block sizes, more alternative routes and fewer dead-ends and cul-dec-sacs^12, 13, 15^. Few residential relocation studies have estimated the extent to which changes in street connectivity are associated with changes in physical activity^8–10^. For instance, in one study from Finland, adults who relocated to a neighbourhood with higher street connectivity (i.e., 3-way or more intersections within 1 km of home) had an increased likelihood of both walking and cycling at least 4-times per week^17^. In the US, women relocating to a neighbourhood with higher connectivity (i.e., fewer cul-de-sacs within 400 m of home) undertook 5000 steps more per week than those who relocated to a neighbourhood with lower street connectivity. In another US study, small but significant increases in self-reported transport, but not leisure, walking minutes was associated with increases in connectivity (ratio of network area to Euclidean buffer area within 1.6 km of home), although not all participants relocated^18^. There was no association between changes in street connectivity (number of intersections per kilometre of road within 1 km of home) and accelerometer-measured weekly minutes of moderate-to-vigorous physical activity (MVPA) or steps in a UK study^19^. Similarly, no association was found between street connectivity (ratio of 3-or more way intersections over 1.6 km area from home) and self-reported neighbourhood-based leisure walking following residential relocation in an Australian study^20^. The mixed findings reflect differences in follow-up times, time exposed to the new built environment, physical activity measurement and definitions, and sample characteristics. The mix of findings also reflects the differences in operational definitions of street layouts, despite all studies using street connectivity to estimate street layouts.

Built environment variables, including street connectivity, estimated in the studies to date do not truly reflect the configuration and topological structure of urban form and street layout. Space syntax theory focuses on the relational aspect of urban form by taking into account the topology of street layouts^21^. In space syntax, “axial lines” are estimated, representing lines of sight (from a location). The configuration of these axial lines is used to estimate “street integration”^22^. Street integration is a complementary measure of street connectivity that reflects changes in the direction needed to travel from one location to all other locations in a defined neighbourhood area. Traditional street connectivity measures typically include counts or density of 3-way or 4-way intersections that do not account for street configuration and thus do not fully capture the underlying opportunities for human movement (e.g., walking) through the neighbourhood. Street integration captures aspects of neighbourhood connectivity not reflected in the traditional measures of street connectivity^23^. Fewer direction changes reflect a more accessible or integrated network. Despite street integration being estimated from the street network, the space syntax theory of natural movement links this novel measure of connectivity with land use and destinations^23, 24^. Application of space syntax theory in public health research remains novel, and among the few existing studies, several have found associations between street integration and physical activity^5, 23–28^. A meta-analysis which included 14 cross-sectional studies linking street integration with pedestrian movement (e.g., walking trips, pedestrian volume, and pedestrian flow) found small-to-moderate effect sizes^29^. Notably, a recent Canadian cross-sectional study found a positive association between street integration within 1.6 km of home and self-reported weekly neighbourhood-based transport walking minutes and participation in leisure and transportation walking after adjusting for reasons for neighbourhood selection^28^. To date, however, no longitudinal residential relocation studies estimating associations between space syntax measures and physical activity have been undertaken^29^.

Longitudinal study designs that can provide robust causal evidence are needed to develop urban design, transportation planning, and public health policies that have a higher likelihood of success of improving physical activity, health, and wellbeing at the population level^30^. Therefore, our study had two objectives. First, to estimate the differences in time spent undertaking moderate-intensity and vigorous-intensity physical activity, and transportation and leisure walking, between three groups, including non-movers, those relocating to less integrated neighbourhoods, and those relocating to more integrated neighbourhoods. Second, to estimate the relationships between different types of exposure (absolute and relative) to street layout integration and differences in time spent undertaking moderate-intensity and vigorous-intensity physical activity and transportation and leisure walking among movers only.

## Method

### Study and sample design

**Figure 1:**
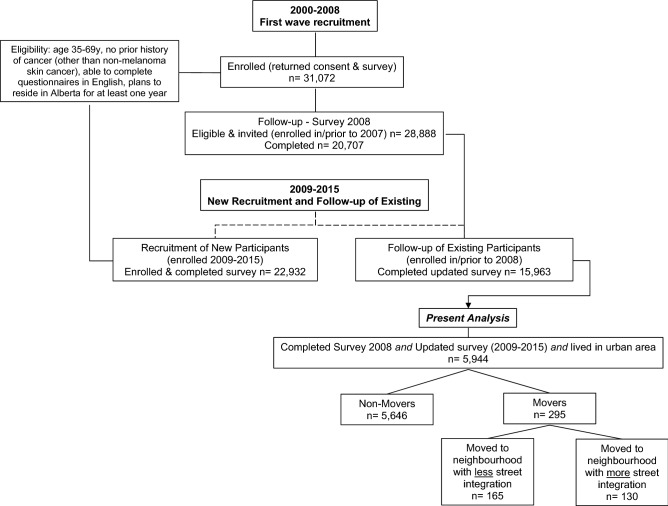
Alberta’s Tomorrow Project (ATP) recruitment.

This study involved a secondary analysis of data from the Alberta’s Tomorrow Project (ATP) and focused on participants living in urban locations. ATP is a longitudinal, province-wide study conducted in Alberta (Canada)^31, 32^. From 2000 to 2008 Albertans aged 35–69 years from urban and rural locations (n = 63,486) were invited via random digit dialing to complete a health and lifestyle (HLQ) of which 31,072 responded (Fig. 1). In 2008, 20,707 of these participants completed a follow-up survey (Survey 2008). Data collection was repeated for a third time between 2009 and 2015, and 15,963 participants returned questionnaires^32^. In the present study, we included participants living in urban locations with complete data for 2008 (herein referred to as “baseline”) and 2009–2015 (“follow-up”) round of questionnaires, where the physical activity questions sufficiently overlapped in wording and formatting allowing them to be compared across these two-time points (2008 versus 2009–2015; n = 5944).

Throughout the ATP data collection, participant residential addresses have been recorded and updated. We took advantage of this reporting by grouping participant’s residential relocation status between 2008 and 2015 into non-movers (n = 5646) and movers (n = 295), and then further subdividing movers into those who relocated to neighbourhoods with less (n = 165) and more (n = 130) street integration. Movers included those relocating within urban areas but excluded those who relocated outside the province or to rural areas. Few residential relocation studies investigating built environments and physical activity include non-movers^18, 33, 34^. Even fewer of these studies^33, 34^ treat non-movers as a non-equivalent comparison group, a study design feature often used in quasi-experiments^35, 36^. Including a non-mover comparison group provides an opportunity to control for and or explain potential factors associated with neighbourhood relocation that are associated with physical activity as well as account for changes in physical activity that may be due to factors others than changes in the neighbourhood environment. The University of Calgary Conjoint Health Research Ethics Board approved the acquisition and analysis of ATP data for this study (REB17-1466). The study was conducted in accordance with the Declaration of Helsinki. All participants provided informed consent when they enrolled in ATP.

### Variables

#### Physical activity

Self-reported physical activity was captured using questions from the International Physical Activity Questionnaire (IPAQ)^37^. The IPAQ provides reliable and valid estimates of different domains of physical activity^37^. At baseline and follow-up, participants reported the number of days in the past week they undertook leisure vigorous-intensity physical activity and leisure moderate-intensity physical activity, leisure walking, and transportation walking for at least 10-min. Participants then reported the time spent on these physical activities during a typical day. We estimated weekly minutes of physical activity by multiplying the number of days doing the activity by the minutes per day for each physical activity. Applying previously used strategies for reducing outliers^38, 39^ physical activities were truncated to 180-min per day.

We estimated seven outcome variables from the physical activity duration data: (1) leisure vigorous-intensity physical activity (VPA); (2) leisure moderate-intensity physical activity (MPA); (3) leisure walking (LW); (4) leisure moderate-intensity physical activity, including leisure walking (MPA + LW); (5) leisure moderate-to-vigorous intensity physical activity including leisure walking (MVPA + LW); (6) transportation walking (TW), and; (7) leisure and transportation walking combined (total walking).

#### Space syntax street integration

**Figure 2:**
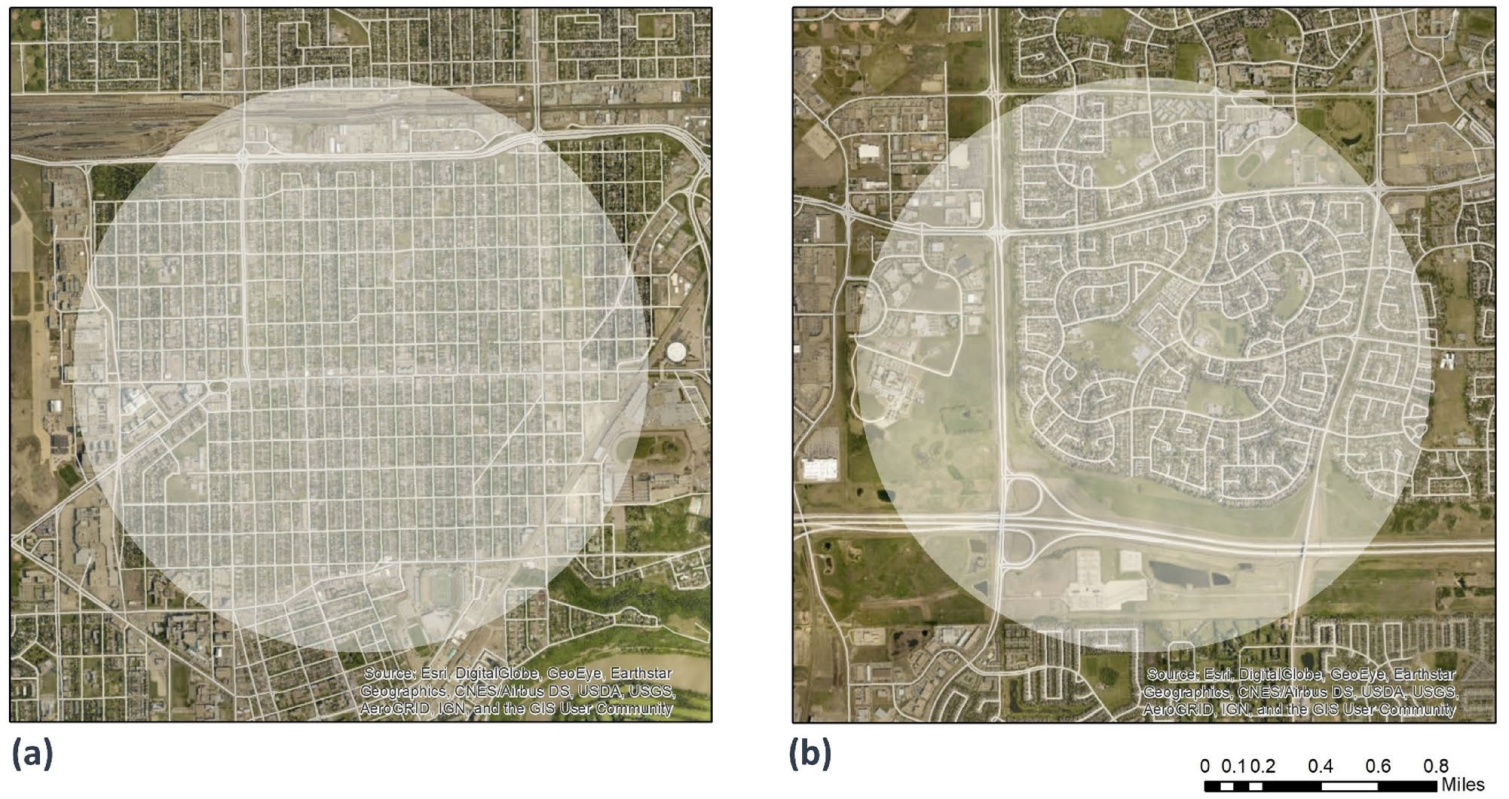
Examples of neighbourhood buffers with (**a**) high street integration (score = 299) and (**b**) low street integration (score = 176) (Imagery Source: Esri, DigitalGlobe, GeoEye, Earthstar Geographics, CNES/Airbus DS, USDA, USGS, AeroGRID, IGN, and the GIS User Community. Figure generated using ArcMap Version 10.3.1).

All Alberta urban 6-digit postal codes in 2008 were geo-located (DMTI Spatial Inc.). Using ArcGIS Pro’s ‘Buffer analysis’ tool, a 1.6 km Euclidian (radial) buffer was created for each postal code. This buffer size reflects the neighbourhood geographical area that is within a 15-min walking distance from home^40, 41^. Using Axwoman^42^ and DepthMap^43^ software, we calculated street integration from street centerline data^44^ derived from the CanMap Streetfiles and Route Logistics data files (DMTI Spatial Inc.)^45^. We estimated a street integration score for each street segment considering all the other street segments within the 1.6 km distance from its centre. Relative to other built environment features, the street layout, and thus intra-neighbourhood connectivity, remains relatively stable over time^18, 19^. A Canadian study demonstrated temporal stability of neighbourhood walkability, population/residential density, street connectivity, and count of retail outlets and services over a 3–7 year period^46^. Thus, the 2008 street integration scores derived from these networks were linked to questionnaire data collected between 2008 and 2009 to 2015, and changes in street integration resulting from residential relocation estimated. We linked street integration scores estimated for 2008 to ATP participant’s 6-digit postal codes of their residential addresses at baseline and follow-up. Approximately 88% of geocoded Canadian postal code locations are within 200 m of geocoded household street addresses^47^. Figure 2 shows an example of neighbourhoods with high (a) and low (b) street integration.

Using baseline and follow-up (post-move) street integration, we estimated one categorical and three continuous exposure change variables. The categorical variable included three groups: (1) non-movers, (2) movers to less integrated neighbourhoods, and; (3) movers to more integrated neighbourhoods. We estimated *absolute* difference in exposure by subtracting baseline street integration from follow-up integration. We estimated *relative* difference in exposure by subtracting baseline street integration from follow-up integration and dividing the difference by baseline integration. Due to the small sample of movers, we did not undertake sensitivity analysis to ascertain the lowest level of change in street integration required to modify physical activity – thus, we considered any difference in street integration resulting from relocation as a change in exposure.

#### Sociodemographic characteristics (covariates)

Baseline sociodemographic variables included sex, age, number of children < 18 years of age, educational attainment, annual gross household income, marital status, and employment status. We also included the elapsed time between the completion of the baseline and follow-up questionnaires for each participant.

### Statistical analysis

Descriptive statistics (mean, standard deviation, and frequencies) and inferential statistics (Welch’s One-way Analysis of Variance with Dunnett’s T3 post hoc comparisons and Pearson’s chi-square with the z-test pairwise comparison of proportions) estimated the differences in baseline sociodemographic and physical activity variables between the three residential relocation groups (i.e., non-movers, movers to lower integration and movers to higher integration). Dependent t-tests estimated the differences in baseline and follow-up physical activity and street integration within residential relocation groups. ANOVA (with least significant difference tests) estimated the differences in street integration (baseline, follow-up, absolute, and relative exposure) between the residential relocation groups.

Our modelling approach was similar to other studies where follow-up physical activity is regressed on baseline physical activity to account for the relationship between baseline and follow-up physical activity^19^. We used multivariable linear regression to regress follow-up minutes on baseline minutes of physical activity adjusted for elapsed time between surveys. By adjusting for elapsed time between surveys, we assumed the adaptation lag was the same for movers regardless of a change in street integration. We saved the unstandardized residuals from the regression models for each physical activity for use as outcomes in subsequent models. Using covariate-adjusted linear regression models, we estimated the mean differences and 95 per cent confidence intervals (95CI) in residualized follow-up physical activity minutes between the three residential relocation groups (non-movers as the reference group). In separate models, we estimated beta slope coefficients (b) and 95CIs between absolute and relative street integration exposures and residualized follow-up physical activity minutes (adjusting for baseline covariates). All inferential statistical tests were two-tailed and statistical significance was set at p < 0.05. Analysis was undertaken using SPSS Statistics for Windows (IBM Corp., Version 25.0., Armonk, NY, USA).

## Results

### Sample characteristics

The majority of participants were female, had completed post-secondary education, married, and employed (Table 1). Non-movers were significantly (p < 0.05) older (55.7 years) than movers to less integrated (51.8 years) and more integrated neighbourhoods (51.8 years) (Table 1). Compared with non-movers, movers to less integrated neighbourhoods included a significantly lower proportion of married individuals (73.3% vs. 60.0%). Compared with non-movers (67.6%), movers to less (77.6%) and more (78.5%) integrated neighbourhoods included significantly higher proportions of employed individuals. The residential relocation groups were similar on all other baseline sociodemographic characteristics (Table 1). The elapsed time between completion of the baseline and follow-up surveys were significantly (p < 0.05) shorter among non-movers (1.5 years) compared with the two groups of movers (1.8 and 1.7 years, respectively).

**Table 1:** Sample characteristics by neighbourhood street integration group (n = 5944).

Baseline sociodemographic	Neighbourhood relocation		
	Non-mover (no change in integration)	Moved to less integrated neighbourhood	Moved to more integrated neighbourhood
	Estimate	Estimate	Estimate
n	5646	165	130
Sex (female %)	61.6	66.7	63.8
Age (mean, [SD])	55.7 (9.1)^a,b^	51.8 (8.7)^a^	51.8 (8.7)^b^
Number of children (mean, [SD])	0.5 (0.9)	0.5 (1.0)	0.5 (0.9)
**Education attained (%)**			
High school or less	17.9	21.8	15.4
Some post secondary	23.6	20.0	26.2
Completed post secondary	58.4	58.2	58.5
**Annual household income (%)**			
≤ $49,999	17.1	24.2	21.5
$50,000 to 99,999	30.6	30.9	26.9
$100,000 to 149,999	23.9	17.6	26.2
$150,000 to 199,999	10.6	7.3	11.5
≥ $200,000	10.2	13.9	10.8
Don’t know/refused	7.5	6.1	3.1
**Marital status (married/defacto %)**	73.3^a,b^	60.0^a^	67.7
**Employment status (employed %)**	67.6^a,b^	77.6^a^	78.5^b^
**Physical activity (mean, [SD]) minutes per week**			
Baseline leisure VPA	67.3 (126.8)	62.9 (122.0)	72.6 (142.8)
Follow-up leisure VPA	69.9 (130.8)	61.5 (150.5)	78.8 (125.8)
Baseline leisure MPA	51.4 (123.0)^a^	52.2 (143.0)	25.4 (60.3) *^,a^
Follow-up leisure MPA	52.5 (124.1)	36.1 (90.8)	61.0 (134.0)
Baseline LW	123.8 (179.1)*	103.8 (155.1)	98.5 (160.7)
Follow-up LW	113.2 (166.5)	89.6 (143.3)	127.8 (201.7)
Baseline leisure MPA + LW	176.6 (243.5)*^,a^	156.0 (215.6)	128.7 (212.9)*^,a^
Follow-up leisure MPA + LW	166.6 (233.0)	125.6 (192.4)	188.9 (251.2)
Baseline leisure MVPA + LW	244.1 (292.6)	218.9 (263.8)	201.3 (267.6)*
Follow-up leisure MVPA + LW	236.9 (290.5)^a^	187.7 (256.8)^a,b^	267.7 (300.0)^b^
Baseline TW	117.3 (178.7)	118.0 (187.3)	117.8 (181.6)
Follow-up TW	119.0 (188.1)	94.6 (171.1)	119.0 (196.2)
Baseline total walking (LW + TW)	242.7 (284.5)*	223.1 (265.5)	221.2 (292.5)
Follow-up total walking (LW + TW)	233.6 (284.9)^a^	184.1 (251.8)^a^	246.8 (314.7)
**Years between surveys (mean[SD])**	1.5 (0.7)^a,b^	1.8 (0.8)^a^	1.7 (0.7)^b^

MPA: moderate-intensity physical activity. VPA: vigorous-intensity physical activity. MVPA: moderate-to-vigorous intensity physical LW: leisure walking. TW: transportation walking. Differences in continuous variables by neighbourhood relocation compared using Welch’s ANOVA with Dunnett T3 post hoc tests. Differences in categorical variables by neighbourhood relocation compared using Pearson’s chi-square.

Same superscript (^a,b^) represents statistically significant (p < .05) difference between neighbourhood relocation groups. *Statistically significant (p < .05) difference between baseline and follow-up physical activity within neighbourhood relocation group (paired t-tests).

### Physical activity characteristics

Among non-movers, weekly minutes of LW, MPA + LW, and total walking (transportation plus leisure) significantly (p < 0.05) decreased between baseline and follow-up (Table 1). Among those who moved to neighbourhoods with higher street integration, weekly minutes of MPA, MPA + LW, and MVPA + LW significantly (p < 0.05) increased between baseline and follow-up. We found no significant differences in physical activity between baseline and follow-up for those who moved to neighbourhoods with less street integration. Between the three residential relocation groups, we found significant differences in baseline MPA, baseline MPA + LW, follow-up MVPA + LW, and follow-up total walking (Table 1).

### Built environment characteristics

Notably, the baseline street integration values for non-movers were significantly lower compared with those moving to less street integration (184.3 vs. 209.5, p < 0.05), but higher compared with those who moved to higher street integration (184.3 vs. 140.8, p < 0.05) (Table 2). Absolute differences in street integration were of similar magnitude but opposite directions between those who moved to neighbourhood with less (-75.2 units) versus more (77.6 units) street integration (Table 2). Relative exposure was negative among those moving to neighbourhoods with less street integration and positive among those moving to neighbourhoods with higher street integration (-0.4 vs. 1.3, respectively, p < 0.05) (Table 2).

**Table 2:** Baseline, follow-up, and exposure street integration estimates by neighbourhood relocation.

	Non-mover (no change in integration) (n = 5646)	Moved to less integrated neighbourhood (n = 165)	Moved to more integrated neighbourhood (n = 130)
Baseline street integration	184.3 (86.7)^a,b^	209.5 (89.0)*^,a,c^	140.8 (74.2)*^,b,c^
Follow-up street integration	184.3 (86.7)^a,b^	134.4 (83.0)*^,a,c^	218.4 (94.5)*^,b,c^
Relative difference exposure		− 0.4 (0.3)^a^	1.3 (3.8)^a^
Absolute difference exposure		− 75.2 (65.8)^a^	77.6 (66.6)^a^

Same superscript (^a,b,c^) represents statistically significant (p < .05) difference in integration and exposure between neighbourhood relocation groups (ANOVA and Least Significant Difference post hoc tests).

### Adjusted differences in follow-up physical activity by residential relocation group

Adjusting for covariates, compared with non-movers, those who moved to higher street integration undertook 27.3 min/week more LW, 45.7 min/week more of MPA + LW, and 54.4 min/week more of MVPA + LW at follow-up (all p < 0.05, respectively) (Table 3). Despite those moving to less street integration undertaking less physical activity at follow-up (for all outcomes) compared with non-movers, none of the differences was statistically significant. Notably, those moving to less versus more street integration significantly differed (p < 0.05) in terms of their weekly minutes of LW, MPA, MPA + LW, MVPA + LW, and total walking (not shown in table).

**Table 3:** Differences in follow-up weekly physical activity minutes^a^ among movers to lower and higher street integration compared with non-movers.

	Follow-up physical activity minutes per week						
	Leisure VPA	Leisure MPA	LW	Leisure MPA + LW	Leisure MVPA + LW	TW	Total walking (LW + TW)
	b (95CI)	b (95CI)	b (95CI)	b (95CI)	b (95CI)	b (95CI)	b (95CI)
**Neighbourhood relocation group**							
Non-mover (no change in street integration; n = 5657)	0	0	0	0	0	0	0
Mover to less street integration (n = 165)	− 6.3 (− 24.0, 11.3)	− 12.2 (− 30.7, 6.4)	− 10.5 (− 34.7, 13.6)	− 23.4 (− 57.1, 10.2)	− 28.8 (− 69.0, 11.4)	− 18.4 (− 45.8, 9.0)	− 29.2 (− 69.2, 10.9)
Mover to more street integration (n = 130)	4.8 (− 14.9, 24.6)	18.2 (− 2.9, 38.9)	27.3(0.2, 54.4) *	45.7(8.0, 83.4) *	54.4 (9.3, 99.4) *	4.7 (− 25.9, 35.4)	30.8 (− 14.1, 75.6)

Unstandardized beta (b) coefficients represent mean difference in physical activity relative to non-movers, adjusted for baseline sex, age, children, education, income, and employment status.

MPA: moderate-intensity physical activity. VPA: vigorous-intensity physical activity. MVPA: moderate-to-vigorous intensity physical LW: leisure walking. TW: transportation walking.

*Statistically significant difference (p < .05) in follow-up physical activity minutes among movers to less and more street integration compared with non-movers (reference group).

^a^The follow-up physical activity variables are unstandardized residuals estimated from a linear regression adjusting for baseline physical activity and elapsed time between completed baseline and follow-up surveys.

### Associations between street integration exposure and follow-up physical activity among movers

Adjusting for covariates, among movers, a one-unit increase in the relative difference exposure in street integration was associated (p < 0.05) with a 7.5 min/week increase in LW at follow-up (Table 4).

**Table 4:** Association in follow-up weekly physical activity minutes^a^ in relation to relative difference and absolute difference exposure to street integration among movers only (n = 295).

	Follow-up physical activity minutes per week						
	Leisure VPA	Leisure MPA	LW	Leisure MPA + LW	Leisure MVPA + LW	TW	Total walking (LW + TW)
	b (95CI)	b (95CI)	b (95CI)	b (95CI)	b (95CI)	b (95CI)	b (95CI)
**Street integration**							
Relative difference exposure	− 1.6 (− 7.1, 4.0)	− 2.6 (− 7.33, 2.22)	7.5 (0.4, 14.6)*	5.0 (− 4.1, 14.2)	3.6 (− 7.7, 14.9)	0.2 (− 7.5, 7.9)	8.04 (− 3.6, 19.7)
Absolute difference exposure	− 0.0 (− 0.2, 0.1)	0.1 (− 0.0, 0.2)	0.1 (− 0.1, 0.3)	0.2 (0.0, 0.5) *	0.2 (− 0.0, 0.5)	0.1 (− 0.1, 0.3)	0.3 (− 0.4, 0.6)

Unstandardized beta (b) coefficients (slope) adjusted for baseline sex, age, children, education, income, and employment status. A positive b reflects a decreasing difference in negative exposure and increasing difference in positive exposure is associated with more physical activity. A negative b reflects a decreasing difference in negative exposure and increasing difference in positive exposure is associated with less physical activity.

Relative difference exposure = [Street integration (follow-up) – street integration (baseline)] / street integration (baseline).

Absolute difference exposure = [Street integration (follow-up) – street integration (baseline)].

MPA: moderate-intensity physical activity. VPA: vigorous-intensity physical activity. MVPA: moderate-to-vigorous intensity physical LW: leisure walking. TW: transportation walking.

*Statistically significant (p < .05) association between street integration exposure and follow-up physical activity.

^a^The follow-up physical activity variables are unstandardized residuals estimated from a linear regression adjusting for baseline physical activity and elapsed time between completed baseline and follow-up surveys.

## Discussion

Our study provides rigorous longitudinal evidence, for the first time, demonstrating that compared with non-movers, people who relocate to neighbourhoods with higher street integration increase certain types of physical activity, including LW, leisure MPA, and leisure MVPA + LW. Among movers, a relative change in street integration exposure following relocation was also positively associated with leisure walking. This finding suggests that moving to a neighbourhood with higher street integration (relative to the pre-move neighbourhood) is more supportive of leisure walking. It also suggests that if someone is moving to a neighbourhood with less street integration, it is better if the difference in the pre and post street integration is minimized.

Our findings support previous longitudinal evidence suggesting that people who relocate to neighbourhoods with more supportive built environment features increase their physical activity^9^, which can potentially provide health benefits^48, 49^. Specifically, our findings support other prospective longitudinal studies showing that increases in street connectivity, another measure of street layouts, can positively affect physical activity, and in particular, walking^17, 18^. Our study contributes to previous evidence regarding connectivity^9^, space syntax^29^ and physical activity by demonstrating longitudinal changes in the environment and behaviour, by examining multiple physical activity outcomes, and by incorporating a non-equivalent comparison group of non-movers.

We found that street integration was temporally positively associated with more leisure walking, MPA, and MVPA. In support of cross-sectional findings from Canada^28^ and US^26^ demonstrating positive associations between space syntax measure of street integration and leisure walking, our study found longitudinal associations between street integration and leisure physical activity. The association between leisure physical activity and street integration is noteworthy given that the conceptual and operational definitions of this and other connectivity indicators tend to relate more to travel ease and accessibility and the ability to reach destinations (i.e., facilitating transport-related physical activity)^23, 24^. Much of the previous evidence regarding associations between space syntax street integration and physical activity suggests that integration is often more supportive of transport walking^5, 23–29^. Notably, Baran et al.^26^ found that access to streets that are necessary to access other local streets (permeability or local connectivity) and global integration (level of access to a street from all other streets) to be positively associated with leisure walking trips. In a Canadian sample, Shatu et al.^50^ found that route distance and direction explain over half of the variance in pedestrian route choice. Others note that the availability of commercial destinations mediates the relationship between street integration and transportation walking^27^. However, studies have yet to examine the extent to which destinations mediate street integration and leisure walking or other types of physical activity. Moreover, other neighbourhood built characteristics might inform route selection (e.g., sidewalk characteristics, amenities, available destinations, traffic, and crowdedness)^51–54^ as well as physical activity^2–10^ that we did not account for in our study. We are unable to say whether higher integrated neighbourhoods in our study also offered more or fewer destinations; destinations are important for supporting transportation walking^3–7^, thus is it difficult to speculate as to why change in street integration was not associated with transportation walking. Nevertheless, our findings suggest that having higher street integration provides more route options, short-distances, and interesting environment in which to undertake leisure walking or other types of physical activity.

Our finding that changes in relative levels of street integration in relation to leisure walking following residential relocation is noteworthy. Exposure to larger increases in street integration could lead to larger increases in leisure walking. For instance, we found that each percentage point increase in street integration following relocation, relative to the pre-move neighbourhood, resulted in about a 7-min per week gain in leisure walking. Individuals gain health benefits even with small increases in physical activity^48, 55^, and although small, these positive shifts in physical activity across many people could have a significant positive impact on population health^56, 57^. This is important to note because the choice of neighbourhood (either with higher or lower street integration) has the potential to widen inequalities in physical activity and health^58, 59^. While yet to be tested, public health and urban design strategies that increase awareness about the importance of neighbourhood design on health (e.g., via media and education, consumer information, economic incentives)^56^ could nudge people to relocate to physical activity supportive neighbourhoods. Urban design and transportation policies that result in the provision of sufficient street integration and other supportive built characteristics in existing and new neighbourhoods might encourage physical activity.

Despite the novel approach to examine street layout and the longitudinal design, our study has several limitations. Our study relied on self-report physical activity data, which may be subject to recall and memory bias^60, 61^ and which were not context (i.e., neighbourhood) specific^28, 61, 62^ potentially underestimating the association between street integration and physical activity. Despite controlling for sociodemographic characteristics and pre-move physical activity and finding similar observed characteristics as non-movers, movers to higher street integration, and movers to less street integration, we did not have access to information about people’s reasons for moving neighbourhood or preferences of neighbourhood built characteristics (i.e., residential self-selection factors). Other individual (e.g., health or weight status or access to a motor vehicle) and neighbourhood characteristics (e.g., changes in destinations or population density) not included in our analysis may have influenced our findings. Moreover, it is possible that participants who relocated within the same postal code were misclassified as non-movers, however, this information was not available. There was also a small possibility that the exposure measure was not well-linked to the home address of participants. Future residential relocation studies should consider including reasons for neighbourhood selection as well as explore associations between the built environment and physical activity among specific population subgroups (gender, socioeconomic status, age, ethnicity etc.). Moreover, information for time-varying covariates was not included given the short amount of elapsed time between collection baseline and follow-up data. We adjusted for elapsed time between the baseline and follow-up survey; however, this does not accurately reflect the amount of time nor exposure to the previous and post-relocation neighbourhood. It is also possible that changes in physical activity in response to changes in the built environment could be delayed or lagged, thus different relationships might be observed in studies where participants have resided in their new neighbourhood for shorter and or longer time periods. Our study did not include other neighbourhood built environment characteristics that are potentially associated with street integration and physical activity^2–10^. Our preliminary analysis included season in which participants completed the questionnaires; however, we excluded this covariate from the final models as it did not affect the estimates.

To expand the evidence on urban design and public health, researchers of this topic should consider using built environment measures that are less data-dependent and are replicable in and comparable across different contexts^63–66^. Space syntax is a useful approach for estimating neighbourhood urban form in exploratory, modelling, and simulation studies that can produce findings to inform urban and transportation policy^63, 67, 68^. Street integration estimates are translatable into policy and practice and only require street or movement networks to estimate, allowing for comparability between different street patterns at neighbourhood and other geographic scales.

Modifying the built environment can impact the health of individuals^69^. Our findings add to the accumulating evidence demonstrating potential causal links between the neighbourhood built environment and physical activity. Here, increased exposure to space syntax integration following residential relocation was associated with increased weekly minutes of leisure walking, MPA + LW, and MVPA + LW. Even small increases in physical activity confer health benefits; thus, improving space syntax integration through better urban planning and design could have a significant positive impact on physical activity on a population scale. Making street integration and other estimates of the built environment available for public access and use could help inform residential relocation choice decisions and achievement of desired levels and types of physical activity.

## Data Availability

The Alberta Tomorrow Project data that support the findings of this study are available from the Alberta’s Tomorrow Project (https://myatp.ca/) following data requisition approval.
